# Evaluation of the pediatric antiretroviral therapy service in Gondar city public health facilities—A case study design with mixed methods

**DOI:** 10.1371/journal.pone.0279890

**Published:** 2022-12-30

**Authors:** Abrham Azanaw, Melaku Birhanu Alemu, Mezgebu Yitayal, Andualem Yalew Aschalew

**Affiliations:** 1 Student Clinic, Student Service Directorate, University of Gondar, Gondar, Ethiopia; 2 Department of Health Systems and Policy, Institute of Public Health, College of Medicine and Health Sciences, University of Gondar, Gondar, Ethiopia; Oregon State University, UNITED STATES

## Abstract

**Background:**

The pediatric antiretroviral therapy (ART) service is introduced to save lives, restore mental and physical functions, and improve the quality of life of children living with HIV/AIDS. This evaluation aimed to assess the implementation status of the pediatric ART service provision in Gondar city administration health facilities to promote evidence-based decision-making for program improvement.

**Methods:**

An institutional-based single case-study design with concurrent mixed methods were applied. The service was evaluated by the availability of essential resources, compliance of health providers with the standard guideline, and caregivers’ satisfaction dimensions. Document review, key informant interviews, observations, and interviewer-administered exit-interview were conducted. The quantitative data were analyzed in descriptive and analytical, while the qualitative data were transcribed, translated, and thematically analyzed. A logistic regression analysis was performed to identify factors associated with caregivers’ satisfaction.

**Results:**

The overall implementation of pediatric ART service was 75.32%. The availability, compliance, and satisfaction were 68.96%, 74.44%, and 84.64%, respectively. Trained healthcare professionals, essential ART drugs, registers, and basic laboratory diagnostic equipment were reasonably available. However, the lack of opportunistic infection medications and adequate rooms were significant gaps in service provision. Respondents noted a shortage of drugs and rooms for consultation and service provision. Short travel distance (AOR = 2.87), low viral load (AOR = 3.15), and sex of caregivers (AOR = 4.98) were significantly associated with good satisfaction.

**Conclusions:**

The overall implementation of pediatric ART service is well based on the pre-determined judgment criteria. The health facilities and policymakers are advised to focus on availing medications to treat opportunistic infections and expanding the health facility to have enough space for consultation and service provision. Furthermore, particular emphasis should be given to caregivers who come from long distances and patients with a high viral load to increase caregivers’ satisfaction.

## Background

Globally, approximately 38 million people were living with the human immunodeficiency virus (HIV) in 2019. Of these, 36.2 million were adults, and 1.8 million were children (<15 years old). Most people living with HIV are from low-and middle-income countries: 20.7 million people (54%) in eastern and southern Africa. The number of people who died of AIDS has declined over the years due to improving access to Antiretroviral therapy (ART) [1].

ART is the daily use of a combination of medicine called "HIV regimen" to stop the virus from multiplying and can suppress it to undetectable levels in the blood [2]. The initial standard treatment for people newly diagnosed with HIV generally includes three antiretroviral drugs from at least two different HIV drug classes.

HIV treatment access is key to the global effort to end acquired immunodeficiency syndrome (AIDS) as a public health threat. By the end of 2019, 25.4 million people with HIV (67%) were accessing ART globally. The Joint United Nations Programme on HIV/AIDS (UNAID) planned that 90% of all people who know their status should be on ART [1, 3]. However, 12.6 million people did not access the treatment. According to the 2018 estimate, 613,000 Ethiopians were living with HIV. However, only 436,000 individuals have received the treatment [4].

Ethiopia is a low-income country experiencing a high number of patients with HIV/AIDS. In response to HIV/AIDS, Ethiopia introduced the ART program to save lives, restore mental and physical functions, and improve the quality of life of people living with HIV/AIDS. The first adult treatment guideline was issued in 2003 and revised in 2005, 2007, 2014, and 2018. A pediatric treatment guideline was also developed and consolidated with the adult guidelines in 2007 [4].

The availability of HIV-related preventive care and treatment services is essential to reduce the burden of the disease. The treatment is aimed at early case detection through testing services, care, support, adherence surveillance, and monitoring. However, many resource-limited countries face challenges in availing of services [5, 6].

A well-functioning ART center needs a separate area for advisory services with appropriate privacy rooms. In India, the assessment of ART centers showed less than half of the facilities (48%) had ideal counseling areas and education and communication materials [7]. The study on the availability of HIV testing, care, and treatment in Ghana revealed viral load and early infant diagnosis, and laboratory testing services were reported at 5.8% and 13.4%, respectively. Shortage of test kits, drugs, and job training were the challenges of various facilities [8]. The study conducted in South Africa showed that the service quality of ART clinic counselors often fails to address HIV prevention. The healthcare provider performed TB symptom screening for only 41.1% of visitors [9].

A study from the 2014 Service Provision Assessment (SPA) survey revealed the capacity of health facilities for HIV/AIDS care was poor. For instance, out of the 873 health facilities, 32.6%, 53.7%, and 74.5% of health facilities providing ART, prevention of mother-to-child transmission (PMTCT), and HIV counseling and testing (HCT) service, respectively. The overall capacity scores were 45% for health centers and 77.1% for hospitals. Nearly one-third and a quarter of the health centers provided HCT and ART services using national guidelines.

Antiretroviral drugs, baseline CD4 count or viral load, and tuberculosis (TB) screening were available in less than 50% of the health centers [10]. Other studies also revealed that inputs for services such as drugs were not available adequately [11, 12]. Moreover, studies on client satisfaction with ART service ranged from 55.2% to 89.6%. Different factors affect client satisfaction, such as income, age, marital status, educational status, distance, occupation, availability of drugs, patient-client interaction, and communication [12–17].

The process evaluation conducted on pediatric ART service in Addis Ababa revealed the overall compliance of healthcare providers to the national guideline of ART service was 81.6% [13]. The evaluation of HIV/ AIDS quality care in Bahir Dar Felege Hiwot referral hospital showed that among patients eligible for ART per the national guidelines, only 205 (76.8%) took ART. In comparison, 34 (12.7%) never started ART, and the remaining 28 (10.5%) failed to continue treatment after initiation [11].

Previous studies only focused on client satisfaction or the availability of materials. A comprehensive and systematic assessment of different program dimensions is essential to make evidence-based decisions for program improvement.

Pediatric antiretroviral treatment failure and viral load are high despite the existing treatment. Therefore, the evaluation of the implementation of pediatric ART services is crucial. As to the authors’ knowledge, there is no evaluation conducted in Ethiopia about pediatrics’ ART implementation status in the health center (HC) setup. Therefore, this evaluation is designed to assess the availability of resources required to provide pediatric ART services effectively, assess the compliance of HCPs with the national guideline in delivering pediatric ART services and determine the level of caregiver satisfaction with ART services provided and identify factors associated with the satisfaction of caregivers/mothers. Moreover, the evaluation results could be used for pediatric ART service improvement and to make evidence-based decision-making. The evaluation’s finding is significant to the public, government, and stakeholders.

## Methods

### Evaluation design and setting

An institution-based case study evaluation design with a concurrent mixed method was used to evaluate the process of pediatric antiretroviral therapy service implementation in Gondar city administration health centers (GCAHCs). The design allows for investigating a contemporary phenomenon in depth and within its real-life context through multiple sources of evidence. The city comprises six sub-cities, 25 kebeles, one specialized hospital, and eight health centers. The total number of ART users is 3,742. The evaluability assessment was conducted from December 04 to 10, 2019, and then the actual evaluation was conducted from February to April 2020.

### Evaluation dimensions and focus

**Fig 1:**
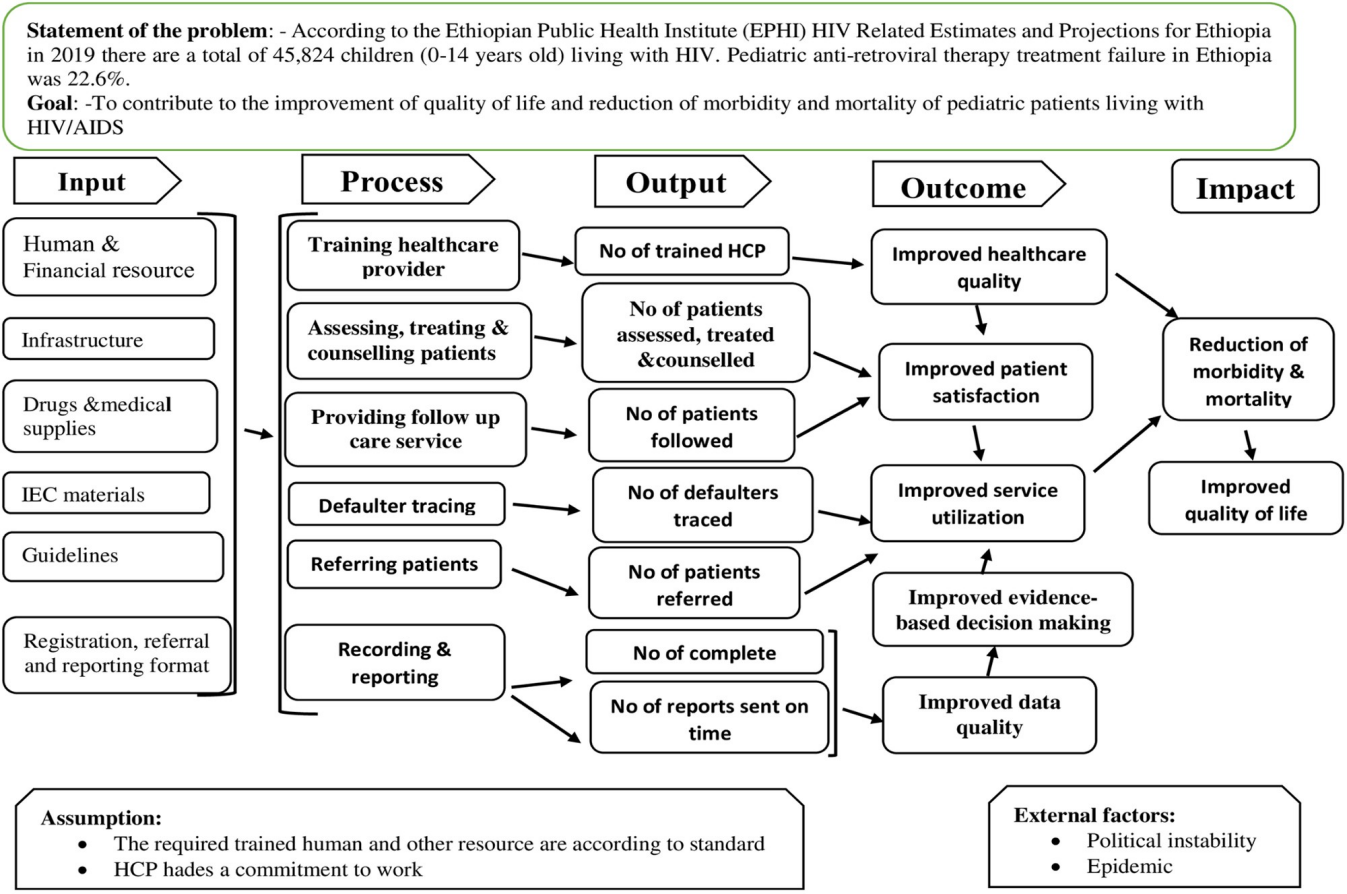
The logic model of the pediatric ART program.

A formative evaluation approach was used to evaluate the process of the pediatric ART program, and three dimensions were used to measure the implementation status of the program. Accordingly, the availability dimension from the access framework [18], the compliance dimension from the implementation fidelity framework [19], and the satisfaction dimension were used. The focus of the evaluation was on the processes of pediatric ART services (Fig 1).

### Population and samplings

#### Population

The source population of this evaluation was program managers, healthcare providers, patients, and medical records. For the satisfaction dimension ART, users were the study population; for the availability dimension ART, the focal person and head of a health center and compliance dimension health care provider (HCP) and patient’s medical record card were considered the study population. For the exit interview, caregivers of children were included, whereas caregivers who visited the health center for the first time were excluded as they may not have adequate prior experience. The head of the health center and ART focal persons were considered the key informants.

#### Sample size

All seven health centers were used to measure the availability of necessary resources in the health facilities. The sample for the satisfaction dimension was calculated using a single proportion population formula from a previous study in the Tigray region [17] by considering a proportion of 89.6%, a 4% margin of error, a 95% confidence level, and a 10% non-response rate. This yielded 246 as a sample size. For the compliance dimension, the above formula was used again by considering the proportion of compliance of HCP for ART guideline 81.0% [20], 5% margin of error, 95% confidence level, and 10% non-response rate. The final sample size was found to be 236.

A total of 14 key informant interviews and 35 observations were conducted. Seven key informant interviews were conducted with the head of health centers and the ART focal person. According to UNAIDS standards tools for evaluation, 3 to 5 direct observations per site are needed to check compliance [21]. Consequently, five observations were conducted in each health center.

### Variables and measurements

The program process was evaluated using availability, compliance, and satisfaction dimensions. The availability of resources was assessed using 14 indicators to determine whether ART drugs, medical supplies, human resources, and logistics (guideline, recording, and reporting tools) required for the program implementation were supplied or not in the health centers. The compliance of healthcare providers was also measured using eight indicators, which measure the adherence level of care providers to the national consolidated guidelines for comprehensive HIV prevention, care, and treatment guidelines during the diagnosis and treatment of children [4]. In addition, the satisfaction was measured using 13 item questions, each containing a five-point Likert scale (1 = strongly disagree, 2 = disagree, 3 = neutral, 4 = agree, and 5 = strongly agree) alternatives. Caregiver satisfaction was classified into two categories, satisfied and dissatisfied, using the demarcation threshold formula [22].

$$\frac{total\;highest\;score-total\;lowest\;score}{2}+\;total\;lowest\;score$$

The dimensions were judged as >85% = excellent, 70–85% = very good; 55–69% = good; 40–54% = fair and <40% = critical.

### Data collection tools and procedures

The resource inventory tool was used to collect data on the availability of program resources to deliver ART services. The questionnaire was adapted from the Ethiopian national guidelines of ART [23].

Documents were reviewed to evaluate healthcare provider’s compliance with the national HIV treatment guidelines. Moreover, observation was undertaken to strengthen the compliance and availability dimensions. The selected indicators were again reviewed with stakeholders for their local relevance.

A structured interviewer-administered questionnaire containing information on caregivers’ sociodemographic characteristics, clinical variables of children, and caregivers’ satisfaction with ART service was used. The tool was developed by reviewing different literature [15, 24].

Indicators were developed by reviewing national guidelines for the treatment of patients with HIV/AIDS in Ethiopia [4]. The selected indicators were again reviewed with stakeholders for their local relevance.

The questionnaire was initially prepared in English and then translated to Amharic, the local language, and back to English to maintain consistency. Six BSc degree clinical nurses and three BSc degree graduate public health professionals were recruited for data collection and supervision, respectively. Two days of training were given on the evaluation objectives, data collection tools, and data collection and supervision techniques. A pretest was conducted out of the study area (at Maksegnit health center), and the necessary correction was made. During observation, the first three observations were discarded to minimize Hawthorn’s effect.

### Data management and analysis

The quantitative data were checked for completeness; codes were given to each questionnaire and entered into Epi Info Version 7 Software. The analysis was conducted using SPSS version 20. Descriptive results were presented using frequencies, percentages, means, and standard deviations. Moreover, a binary logistic regression analysis was done to identify satisfaction-related factors. Variables with a p-value of less than 0.25 were a candidate for the multivariable logistic regression. A P-value of <0.05 in a multivariable logistic regression was used to declare factors associated with caregivers’ satisfaction. The Chi-square assumption and model fitness were checked using the Hosmer and Lemeshow goodness of fit test. Qualitative data was gathered, transcribed, and translated into English, and thematic analysis was done. Finally, the result was judged based on the judgment matrix.

### Ethics approval and consent to participate

This study was conducted in compliance with the Helsinki declaration. Ethical approval was obtained from the Ethical Review Board of the Institute of Public Health, College of Medicine and Health Science, University of Gondar (Ref No/IPH/837/6/2020). A permission letter was obtained from the Gondar City Administration Health Office. All study participants were oriented on the objectives and purpose of the study before participation. In addition, the confidentiality and anonymity of the participants were assured. Participation in the survey was voluntary, and individuals were free to withdraw or stop the interview at anytime. After ensuring the confidentiality nature of responses, written informed consent was obtained from each participant’s parent or guardian before data collection. An assent was taken from a child’s affirmative agreement to participate in research from all children 12 years of age and above, in addition to the consent of a parent or guardian.

## Results

### Availability of resources

Gondar city has seven health centers that provide comprehensive ART services. Among 311 healthcare providers, only 51 have ART training. Six of the seven HCs had staffed with trained health professionals in the treatment and counseling service area.

### ART and OI drugs

The first-line ART drugs for the age group 10–18 years were available in all HCs. The first-line drug for 3–10 years’ treatment was only available in 71% of the HCs. However, Lopinavir (LPV/r), the first-line drug to treat children younger than three years, was unavailable in all HCs. In addition, medications for the treatment of OI were not available satisfactorily or at all (Table 1).

**Table 1:** Summary of ART resource availability indicators in the last three months in GCAHCs, April 2020.

Indicators	E*	O*	W*	S*	A*	JP*
Proportion of health centers with ART implementation guideline at the ART clinic pharmacy and laboratory room on the day of data collection time.	7	3	1.8	0.77	42.77	Fair
Proportion of health center with Amoxicillin drug	7	3	3	1.28	42	Fair
Proportion of health center with pediatric weight scale on the day of data collection time	7	7	1.5	1.5	100	Excellent
Proportion of health center with pediatric Co-trimoxazole prophylaxis	7	3	2.1	1.28	60	Fairly
Proportion of health center with private counselling room	7	0	2.1	0	0	Critical
Proportion of health center with non-stretchable plasticized tape meter on the day of data col0.lection time	7	3	0.9	0.5	55	Good
Proportion of health centers with trained health professionals as per the guideline.	7	6	1.2	1.02	85	Very good
Proportion of health centers with a growth monitoring chart on the day of data collection time.	7	5	3	2.14	71	Very good
Proportion of health center with pediatrics strength of AZT/3TC/EFV	7	5	3	2.14	71	Very good
Proportion of health center with pediatrics strength of AZT/3TC/ NVP	7	7	3	3	100	Excellent
Proportion of health center with pediatrics strength of TDF / 3TC/EFV	7	7	3	3	100	Excellent
Proportion of health center with pediatrics strength of ABC/3TC/EFV	7	5	3	2.14	71	Very good
Proportion of health centers with separate case manager room	7	1	3	0.42	14	Critical
Proportion of health centers with HIV test kit like stat-pak, Abon and SD-Bioline–tie breaker	7	7	1.5	1.5	100	Excellent
Pediatric ART service overall process for availability sub-dimension			30	20.69	68.96	Good

E* = Expected, O* = Observed, W* = weight, S* = Score ((observed X weight)/Expected), A* = Achievement in percentage ((S/W) * 100)

JP* = Judgment Parameter (>85% = excellent, 70–85% = very good; 55–69% = good; 40–54% = fair and <40% = critical)

AZT, Zidovudine; 3TC, Lamivudine; EFV, Efavirenz; NVP, Nevirapine; TDF, Tenofovir

### Infrastructure and laboratory

All health centers had rooms for ART service delivery. But none of the health centers’ rooms provides privacy for counseling. Stool examination, blood film, and HIV serologic tests were available in all health centers. However, other laboratory tests were scarce (Table 1).

A key informant’s interview showed a shortage of resources to implement pediatric ART services. The ART rooms were not convenient for privacy, and drugs for opportunistic infection treatment were unavailable in all HCs.

"Health center’s setups for ART clinic rooms are not properly settled in addition to the shortage of room to provide service. We are working in one room with two health care providers, and ART and TB services are in one room in which patients with HIV/AIDS might contact patients with TB and are vulnerable to TB disease" [ART focal person].

"Required resources were not sufficient. According to our facility, the availability of the room, different options, and dosage of ART drugs and OI drugs, particularly pediatric OI drug-like co-trimoxazole, were not available [ART focal person].

"NGOs have donated OI drugs supply for the program but not continuously. There is also a shortage of room for ART services like private counseling rooms, which is challenging" [Head of HC].

### Registers and guidelines

Forms, cards, and guidelines were available in all health centers. However, the ART guideline was found in 57% of pharmacies and 42.8% of laboratories.

Based on the judging criteria, the pediatric ART program resource availability was judged as good, 68.96%. But some resources need improvements, such as drugs for treating OI, the privacy of rooms, and training for one health center’s ART focal (Table 1).

### Compliance of healthcare providers with the national ART guideline

A total of 236 client medical record cards were reviewed, and 35 ART care sessions were observed to assess the compliance of ART services with the national guideline. Children started ART based on the national guidelines. TB screening was checked and filled in on all patient’s medical cards. Moreover, approximately 42.5% of treatment regimen change was not written the reason for the change. Out of the 236 patients included in this study, 185 (78.3%) had the most recent CD4 count. The overall compliance of healthcare providers to the national ART guideline was very good (Table 2). The observation result showed that WHO staging and follow-up schedules were carried out appropriately for all clients. But monitoring for ART drug adherence and TB screening was done for 33.3% and 31.4% of children, respectively (Table 3).

**Table 2:** Summary of compliance sub-dimensions indicators in GCAHCs, April 2020.

Indicators	E*	O*	W*	S*	A*	JP*
The proportion of children who measured CD4 count	236	185	7.7	6.0	78.3	Very good
The proportion of children measured viral load as per the guideline	236	203	7.7	6.6	85.9	excellent
A proportion of children had a baseline of CBC before treatment initiation	193	51	3.6	0.9	26.4	Critical
The proportion of pediatric (client) assessed nutritional status.	236	142	4.5	2.7	60.1	Good
The proportion of children screened for TB	236	236	7.7	7.7	100	Excellent
The proportion of patients with previous ARV regimen changes for whom the reason for regimen change is documented	127	73	7.7	4.4	57.5	Good
The proportion of patients on ART who had registered own and contacted address	236	236	2.9	2.7	100	Excellent
The proportion of the patient assessed for drug side effects every visit	236	183	3.2	2.5	77.5	Very good
**Overall compliance of HCP with the national guideline**			**45**	**33.5**	**74.4**	**Very Good**

E* = Expected, O* = Observed, W* = weight, S* = Score ((observed X weight)/Expected), A* = Achievement in percentage ((S/W) * 100)

JP* = Judgment Parameter (>85% = excellent, 70–85% = very good; 55–69% = good; 40–54% = fair and <40% = critical)

**Table 3:** Participatory observation result of healthcare provider performance in GCAHCs, April 2020.

Expected activities performed by HCP	Expected	Performed service	Percent
Does the provider welcome the clients	35	9	25.7
Does privacy maintained	35	24	68.5
Does encourage the client to ask question	35	15	42.8
Check for weight	35	35	100
Monitoring for ARV drugs adherence	35	10	33.3
Counseling/support for adherence	35	25	71.4
Check for nutritional status	35	20	57.2
Prescribing ARV Drug	35	35	100
Requesting for viral load test	5	5	100
TB screening	35	11	31.4
Does advice on diet	35	14	40
Accuracy of assessment and diagnosis (WHO staging)	35	35	100
Does develop an appropriate follow-up schedule	35	35	100

ARV, Antiretroviral

### Satisfaction of caregiver with pediatric ART services

#### Sociodemographic characteristics of participants

A total of 241 caregivers were interviewed, with a 97.96% response rate. The mean age of caregivers was 37.7 ± 8.2 years; out of the participants, almost half (48.5%) were married, and 10.8% were single. More than half (57.26%) of the children were female. The majority, 68.9% and 77.59% of children, had ≤500 CD4 count and ≤1000 copies/ml viral load, respectively (Table 4).

**Table 4:** Characteristics of a study participant in GCAHCs, April 2020.

Variables	Frequency	Percent
**Age of child**		
** <5**	18	7.47
** 6–14**	161	66.80
** 15–18**	62	25.73
**Age of caregiver**		
** 25–29**	39	16.18
** 30–34**	50	20.75
** 35–39**	65	26.97
** 40–44**	43	17.84
** >44**	44	18.26
**Sex of child**		
** Male**	103	42.74
** Female**	138	57.26
**Sex of parent or Caregiver**		
** Male**	44	18.26
** Female**	197	81.74
**Educational status of the respondent**		
** Unable to read and write**	72	29.88
** Primary education**	100	41.49
** Secondary and above**	69	28.63
**Occupational status**		
** Government employee**	40	16.60
** Merchant**	83	34.44
** Daily labor**	62	25.73
** Housewife**	45	18.67
** Others**	11	4.56
**Religion of respondent**		
** Orthodox**	171	70.95
** Muslim**	65	26.98
** Protestant**	5	2.07
**Marital status of the respondent**		
** Married**	117	48.55
** Single**	26	10.79
** Divorced**	41	17.01
** Widowed**	57	23.65
**Reason for a current visit for the child**		
** ART clinic scheduled visit**	144	59.75
** ART clinic unscheduled visit**	97	40.25
**Monthly average income (ETB)**		
** <1200**	71	29.46
** 1200–2400**	65	26.97
** ≥ 2400**	105	43.57
**CD4 count cells/mm3**		
** 201–350 cells/mm3**	32	13.28
** 351–500 cells/mm3**	43	17.84
** ≥ 500 cells/mm3**	166	68.88
**Viral load level**		
** ≤1000 copies/ml**	187	77.59
** > 1000 copies/ml**	54	22.41
**Waiting time**		
** <30 minute**	193	80.08
** ≥ 30 minutes**	48	19.92
**Distance to reach HC**		
** < 60 minutes**	164	68.05
** ≥ 60 minutes**	77	31.95

#### Caregiver satisfaction with pediatric ART services

A high level of satisfaction was observed with the medical records of confidentiality (88.80%) and technical competence of HCP (87.97%). The lowest satisfaction was observed regarding on appropriateness of the waiting area (43.15%). The overall satisfaction of caregivers on ART service was 84.64% (95% CI = 79.48–88.69) (Table 5).

**Table 5:** Caregiver satisfaction on the ART service provided in GCAHCs, April 2020.

No	Variables	Dissatisfied number (%)	Satisfied number (%)
**1**	How satisfied are you with the convenience of the physical setting of the ART service?	91 (37.76)	150 (62.24)
**2**	How satisfied are you with the convenience of opening hours?	34 (14.11)	207 (85.89)
**3**	How are you satisfied with privacy during the examination?	98 (40.66)	143 (59.34)
**4**	How satisfied are you with the technical competence of HCPs?	29 (12.03)	212 (87.97)
**5**	How satisfied are you with the medical records of confidentiality?	27 (11.20)	214 (88.80)
**6**	How satisfied are you with the available drugs at the health center?	89 (36.93)	152 (63.07)
**7**	How satisfied are you with the waiting time to receive ART service?	86 (35.68)	155 (64.32)
**8**	How satisfied are you with the appropriateness of the waiting area?	137 (56.85)	104 (43.15)
**9**	How satisfied are you with the laboratory service?	94 (39.00)	147 (61.00)
**10**	How satisfied are you with the appointment date?	34 (14.11)	207 (85.89)
**11**	How satisfied are you with the explanation given by care providers?	33 (13.69)	208 (86.31)
**12**	How satisfied are you with the courtesy of HCPs?	40 (16.60)	201 (83.40)
**13**	How satisfied are you with the general ART services?	39 (16.18)	202 (83.82)

#### Factors associated with satisfaction of pediatric ART client caregiver

In this study, the sex of caregivers, distance to the health facility, and viral load were significantly associated with caregivers’ satisfaction with ART services.

For caregivers who came for less than 60 minutes to the HC, the odds of satisfaction with ART service were 2.87 (95% CI: 1.19, 6.90) times higher than the odds of caregivers who came for more than 60 minutes to the HC. For caregivers whose child’s viral load was less than 1000 copies/ml, the odds of satisfaction with ART service were 3.15 (95% CI: 1.34, 7.39) times higher than the odds of caregivers whose child viral load was greater than 1000 copies/ml. Moreover, the odds of satisfaction among male caregivers were 4.98 (CI: 1.35, 19.13) higher than the odds of satisfaction among female caregivers (Table 6).

**Table 6:** Factors associated with caregivers’ satisfaction towards pediatrics ART services in GCAHCs, April 2020.

Variables	Satisfaction		COR (95%CI)	AOR (95%CI)
**Sex of parent or Caregiver**	Satisfied	Dissatisfied		
** Male**	41	3	2.87 (0.83,9.74)	4.98 (1.35,19.13)*
** Female**	163	34	1	
**Marital status**				
** Single**	20	6	1	
** Married**	106	11	2.89 (0.95,8.71)	2.17 (0.64,7.38)
** Divorce**	33	8	1.23 (0.37,.04)	0.73 (0.19,2.85)
** Widowed**	45	12	1.12 (0.36,3.42)	0.59 (0.15,2.37)
**Education**				
** No education**	63	9	1	
** Primary**	87	13	0.95 (0.38, 2.37)	0.75 (0.25, 2.21)
** Secondary and above**	54	15	0.51 (0.21, 1.26)	0.39 (0.13, 1.24)
**Distance**				
** < 60 minutes**	147	17	3.03 (1.48, 6.20)	2.88 (1.19, 6.90)*
** ≥ 60 minutes**	57	20	1	
**Waiting time**				
** <30 minutes**	155	23	1	
** ≥ 30 minutes**	49	14	1.92 (0.92, 4.02)	1.12 (0.43, 2.87)
**Viral load**				
** ≤1000 copies/ml**	165	22	2.88 (1.37, 6.06)	3.15 (1.34, 7.39)**
** > 1000 copies/ml**	39	15	1	
**Constant**				1.45 (0.24, 8.65)

AOR, Adjusted odds ratio; COR, Crude odds ratio

*P-value < 0.05,

**P-value < 0.01

The judgment matrix synthesizes all available information aiming to characterize the implementation degree. Accordingly, the overall implementation status was also judged but needed improvement (Table 7).

**Table 7:** Summary of judgment matrix for processes pediatric ART service in GCAHCs, April 2020.

Dimensions	Expected %	Weight %	Observed %	Evaluation rate
**Availability**	100	30	20.69	≥85–100 = Successfully implemented;
**Compliance**	100	45	33.48	70–84% = Fair but need improvement and
**Satisfaction**	100	25	21.15	<70 Needs urgent improvement.
**Total score**			**75.32**	**Fairly implemented but need improvement**

## Discussion

This evaluation evaluates the process of pediatrics ART service at Gondar city health centers using availability, compliance, and accommodation dimensions. The scores of availability, compliance, and satisfaction were 68.96%, 74.44%, and 84.64%, respectively. The overall implementation process was found to be 75.32%, meaning that the program is implemented fairly based on the judgment matrix but needs improvement to bring about the intended effects.

Healthcare resources are understood as key to maintaining and improving health services. Access to healthcare services is also a function of the availability of health services inputs. This evaluation revealed that the HCs fulfilled most of the required inputs for ART services except shortage of some supplies and laboratory. Trained human resources, drugs, rooms, guidelines, and registers were reasonably available. Six of seven HCs were staffed with ART-trained health professionals, and all HCs had room for service delivery. Studies in resource-limited countries showed that lack of healthcare infrastructure, limited availability of pediatric drug formulations, and shortage of workforce expertise in pediatric HIV care are the challenges in resource-limited countries [6]. This evaluation revealed that the workforce is relatively good and that most facilities appointed ART-trained healthcare professionals. Basic laboratory examination facilities, guidelines, and registers were available. However, infrastructure is still the problem. For instance, none of the rooms provided privacy for counseling, and there were no separate spaces for the case manager.

Regarding medications, first-line ART drugs were almost available in all health facilities, except lopinavir. However, Co-trimoxazole prophylaxis and amoxicillin were absent in more than half of the HCs. Studies in Bahir Dar, Ethiopia [11], and South Africa [25] aligned with these findings. This finding is generally lower than the findings with health facilities in Addis Ababa, Ethiopia [26].

Compliance with the guideline and treatment protocol is crucial to address service delivery objectives. And this study revealed that the overall compliance of healthcare professionals was very good (74.44%). This might be related to the availability of health professionals who got training on national consolidated guidelines for comprehensive HIV prevention, care, and treatment in the majority of health centers. This is higher than studies in Bahir Dar [11] and Dessie [27], Ethiopia, which can be explained by the time-to-time emphasis given by the government on the capacity building of healthcare professionals. Conversely, this finding was lower than studies in Bure primary hospital and health facilities in Addis Abebe [26, 28].

Moreover, clinical and laboratory monitoring were good such as viral load. The contradicted finding was seen in TB screening; on document review; all children were screened for TB. However, the observation showed that screening was conducted for only less than one-third of the sessions.

The overall satisfaction of caregivers with ART service was 84.64%. Resource availability and compliance of healthcare providers give implicit evidence for caregivers’ satisfaction. Studies support this at Jimma and Addis Abeba [26, 29]. But higher than the studies conducted in Wollega, Hossana, Midregenet, Gondar, Vietnam, and Lusaka [12–15, 30, 31] and lower than studies in Cameron and South Africa [32–34]. Distance to the health facility, male caregiver, and viral load was significantly associated with ART service satisfaction.

Distance to the health facility is one determinant of satisfaction. Caregivers who come from a lower distance had higher odds of satisfaction with the service than caregivers from a far distance. Studies in other settings also align with this finding [13, 17, 35]. Caregivers of a child with a low viral load had higher odds of satisfaction with ART service than caregivers of a child with a higher viral load. The main goal of HIV service (treatment) is to reduce a patient’s viral load to an undetectable level and is a key indicator of treatment success. Low viral load gives the body’s immune system a chance to fight infections and protect the body. A study that shows an association between viral load and patient satisfaction is spared. However, evidence revealed a significant inverse association between viral load and CD4 count: low viral loads were associated with higher CD4 counts [36] and higher CD4 counts and satisfaction [16]. Moreover, male caregivers had higher odds of satisfaction with ART service than female caregivers. Studies have contradicted findings that [31, 33, 37] females were more satisfied than males, but other results indicated no significant difference between males and females [30].

The possible limitation of this evaluation was the hawthorn effect, which might have occurred during the direct observation of the interactions between ART patients and HCPs. To minimize this, we dropped the first three observations.

## Conclusions

The pediatric ART service is fairly implemented well in Gondar city administrative health centers. However, which needs improvement. The implementation of the services needs improvement. Based on the agreed criteria, the availability and compliance dimensions’ scores were good and very good, respectively. The availability of trained healthcare professionals and ART drugs was good. However, resources such as drugs for treating opportunistic infection; adequate consultation rooms that provide privacy should be available to improve the service. Moreover, particular emphasis should be given to those who come from long distances and patients with a high viral load to enhance their satisfaction with ART service.
